# LncRNA LSINCT5/miR-222 regulates myocardial ischemia‑reperfusion injury through PI3K/AKT pathway

**DOI:** 10.1007/s11239-021-02506-3

**Published:** 2021-06-28

**Authors:** Xueying Tong, Jiajuan Chen, Wei Liu, Hui Liang, Hezhong Zhu

**Affiliations:** 1 Department of Geriatrics, Taihe Hospital, Hubei University of Medicine, No. 32 Renminnan Road, Shiyan City, 442000 Hubei Province China; 2 Department of Cardiology, Taihe Hospital, Hubei University of Medicine, No. 32 Renminnan Road, Shiyan City, 442000 Hubei Province China

**Keywords:** Myocardial ischemia/reperfusion injury, Hypoxia-reoxygenation, lncRNA LSINCT5, miRNA-222, PI3K/AKT pathway

## Abstract

Cardiovascular diseases rank the top cause of morbidity and mortality worldwide and are usually associated with blood reperfusion after myocardial ischemia/reperfusion injury (MIRI), which often causes severe pathological damages and cardiomyocyte apoptosis. LSINCT5 expression in the plasma of MI patients (n = 53), healthy controls (n = 42) and hypoxia-reoxygenation (HR)-treated cardiomyocyte AC16 cells was examined using qRT-PCR. The effects of LSINCT5 on cell viability and apoptosis were detected by MTT and flow cytometry, respectively. The expression of apoptosis-related proteins Bcl2, Bax and caspase 3 were tested by Western blot. The interaction between LSINCT5 and miR-222 was predicted by bioinformatic analysis. Moreover, changes in viability and apoptosis of AC16 cells co-transfected with siLSINCT5 and miR-222 inhibitor after HR treatment were examined. At last, the expression of proteins in PI3K/AKT pathway, namely PTEN, PI3K and AKT, was examined to analyze the possible pathway participating in LSINCT5-mediated MI/RI. Our study showed that LSINCT5 expression was upregulated in the plasma of MI patients and HR-treated AC16 cells. LSINCT5 overexpression significantly decreased cell viability and apoptosis. Luciferase reporter gene assay and RNA pulldown assay showed that LSINCT5 was a molecular sponge of miR-222. MiR-222 silencing in AC16 cells simulated the phenotypes of MIRI patients and HR-treated cells, indicating that LSINCT5 functions via miR-222 to regulate proliferation and apoptosis of HR-treated AC16 cells. We also showed that proteins of PI3K/AKT signaling pathway were affected in HR-treated AC16 cells, and LSINTC5 knockdown rescued these effects. LncRNA LSINCT5 was upregulated during MI pathogenesis, and LSINCT5 regulated MIRI possibly via a potential LSINCT5/miR-222 axis and PI3K/AKT signaling pathway. Our findings may provide novel evidence for MIRI prevention.

**Supplementary Information:**

The online version contains supplementary material available at 10.1007/s11239-021-02506-3.

## Highlights

- LSINCT5 expression is significantly upregulated after myocardial ischemia/reperfusion injury.
- Knocking down of LSINCT5 protects cell model of myocardial ischemia/reperfusion injury from cell death.
- LSINCT5 functions as a molecular sponge of miR-222 to regulate myocardial ischemia/reperfusion injury.
- LSINCT5/miR-222 might regulate myocardial ischemia/reperfusion injury through PI3K/AKT pathway.

## Introduction

Cardiovascular diseases rank the most common and mortal disease worldwide, and their morbidity rate increases with age, with 23 million people die by 2030, as estimated by the World Health Organization [1]. It is notable that the major cause of cardiovascular diseases is myocardial infarction, which is usually initiated by the insufficient blood supply to the heart. Reperfusion strategies, including coronary thrombolysis treatment, percutaneous coronary intervention, and coronary artery bypass surgery, are the current standard therapies to improve myocardial infarction [2]. However, these therapies may result in paradoxical cardiomyocyte dysfunction, known as myocardial ischemia/reperfusion injury (MIRI) [2, 3]. Numerous studies have revealed the potential molecular regulation during MIRI, such as hypoxia-induced reactive oxygen species (ROS) accumulation, inflammatory factors dysregulation, intracellular calcium stress, platelet and neutrophil hyperactivation, activated myocardial innate immune response, and cardiomyocyte cell apoptosis [3, 4]. The primary regulatory pathway PI3K/AKT, which is closely associated with cell proliferation and apoptosis, was also reported to participate in MIRI [5]. However, the mechanisms of MIRI are still unclear. Therefore, researchers have tried their best to explore novel regulatory mechanisms such as noncoding RNAs with the hope of finding effective clinical treatment strategies for MIRI.

Noncoding RNAs (ncRNAs) are key components of the transcriptome and play important roles in both normal biological activities and pathological processes. Current studies of noncoding RNAs are mainly focused on microRNAs (miRNAs) and long noncoding RNAs (lncRNAs) because of their powerful roles in altering gene expression and cell behaviors. MiRNAs are small single-strand RNAs in the length of 20–24 nucleotides that could target and bind to the 3’ UTR regions of mRNAs and post-transcriptionally affect the expression of almost 50% of genes [6]. LncRNAs are a novel family of long noncoding transcripts with the length ranging from 200 to thousands of nucleotides [7]. LncRNAs can form molecular scaffolds to affect chromatin modification, participate in epigenetic regulation, sponge miRNAs, and further regulate the expression of corresponding target genes [8, 9]. Over the past few decades, increasing evidences have revealed a wide range of biological functions of lncRNAs and their aberrant expression in the progression of diverse diseases such as cancer, neurological dysfunctions, metabolic disorders, and cardiovascular diseases [10]. Several studies have put efforts into deciphering the roles of lncRNAs in the generation and development of cardiovascular diseases. It is now well recognized that the aberrant level of lncRNAs is associated with the pathogenesis of myocardial infarction and heart failure [11, 12]. For example, a global transcriptomic analysis of patient cohorts with myocardial infarction indicated that mitochondrial lncRNA LIPCAR was downregulated shortly after myocardial infarction but upregulated at the later stages [13]. Suppression of lncRNA KCNQ1OT1 protected cardiomyocytes from MIRI and associated with p38 signaling [14]. LncRNA long stress-induced noncoding transcript 5 (LSINCT5) is a member of the stress-induced lncRNA family, which was reported to be upregulated in various cancers and involved in cell proliferation and metastasis [15, 16]. For instance, LSINCT5 was indicated to act as an oncogene in osteosarcoma and gastric cancer [17, 18]. Zhu et al. reported that LSINCT5 promoted the progression of bladder cancer via activating Wnt/β-catenin signaling [19]. A recent study revealed the regulatory axis of B-type-natriuretic peptide/LSINCT5/caspase-1/interleukin 1β signaling pathway in myocardial cell apoptosis [20]. However, the specific role of LSINCT5 in cardiovascular diseases has yet not be clarified.

In this study, we aimed to demonstrate the role of LSINCT5 in MIRI and the possible mechanisms involved. We emphatically investigated the potential targets of LSINCT5 and possible regulatory axis and found microRNA miRNA-222 could potentially interact with LSINCT5 and that the regulation of LSINCT5/miRMA-222 on MIRI might through targeting the PI3K/AKT pathway. Our findings provide potential supportive evidence for MIRI therapy.

## Methods

### Cell culture and hypoxia-reoxygenation (HR) model

The AC16 cells were from BeNa Culture Collection (Beijing, China) and maintained in Dulbecco's modified Eagle's medium (DMEM) (#11330032, Gibco, Thermo, Waltham, MA, USA) containing 10% fetal bovine serum (FBS) (#10270-106, Gibco), 100 U/mL penicillin, and 100 μg/mL streptomycin in a 37℃ incubator with a humidified atmosphere of 5% CO_2_.

To construct a hypoxia-reoxygenation (HR) model to mimic MIRI, AC16 cells were cultured to achieve 80–90% confluence, trypsinized, and seeded in 6-well plates at a density of 1 × 10^5^ cells per well. Meanwhile, DMEM media were deprived of glucose and serum, and cells were placed in a low-oxygen atmosphere built by 85% N_2_, 10% H_2_ and 5% CO_2_. After incubated in a low-oxygen atmosphere for 6 h, the medium was replaced with fresh complete DMEM media and cells were placed in a normal 5% CO2 atmosphere to incubate for another 2 h.

### Human specimens

A total of 53 MI patients who were admitted to Taihe Hospital from May 2018 to May 2019 were included in the study if they (a) were diagnosed as MI, (b) had a complete medical record, and (c) completed treatment and the 5-year follow-up. Patients were excluded if they (a) had other severe diseases, (b) failed to complete treatment or follow-up, and (c) died of other causes during the follow-up. The 53 MI patients included 35 males and 18 females and were 37 to 66 years old with a mean age of 49.3 ± 7.2 years old. In addition, 42 healthy volunteers (29 males and 13 females) at the age from 35 to 66 years old with a mean age of 50.2 ± 7.3 were included in the study. No significant differences in age and gender were found between the 2 groups. Blood samples (5 mL) were collected from each patient and volunteer. This study was approved by the Ethics Committee of the Taihe Hospital. All participants signed the informed consent.

### Cell transfection

The sequence of LSINCT5 was obtained from the Pubmed website. The siRNA targeting LSINCT5 and the miR-222 mimic and inhibitor were designed and purchased from Ribobio (Guangzhou, China). AC16 cells were seeded in 6-well plates at a density of 1 × 10^5^ cells per well and incubated overnight to achieve monolayer confluence and transfected with indicated siRNA or miRNA accompanied with Lipofectamine 2000 (#11668019, Invitrogen, Carlsbad, CA, USA) according to the manufacturer’s instruction. The cells were transfected for 48 h and then subjected to subsequent hypoxia-reoxygenation administration.

### MTT assay

AC16 cell proliferation was analyzed using the MTT assay. Cells were seeded into 96-well plates at a density of 1 × 10^3^ cells per well and separately treated as previously described. At the indicated time point, 20 μL of MTT (5 mg/mL) (# 11465007001, Roche, Basel, Switzerland) was added to each well and incubated for another 4 h. Then, the supernatants were removed and 150 μL of DMSO was added to each well to terminate the reaction. At last, the absorbance values at 490 nm were determined using an absorbance reader.

### Detection of myocardial injury markers

Lactate dehydrogenase (LDH) and creatine kinase isoenzyme (CK‑MB) were regarded as biomarkers released by cells subjected to myocardial ischemia. We detected the levels of LDH and CK-MB to measure the degree of AC16 cell damaged by HR treatment. LDH levels were detected using LDH assay kits (#C0016, Beyotime, Shanghai, China) following the manufacturer’s instructions. CK-MB levels were evaluated by ELISA kits (#EHCKMB, Invitrogen, CA, USA), and the absorbance was measured at 450 nm using a microporous plate reader.

### Flow cytometry

Flow cytometry was adopted to detect cell apoptosis. Cells were seeded in 6-well plated, transfected, treated HR, and later collected for dual staining with Annexin V-FITC and propidium iodide following the manufacturer’s protocols (#C1062S, Beyotime, Shanghai, China). Annexin V^−^/PI^−^ cells were living cells, Annexin V^+^/PI^−^ cells were at the early apoptotic stage, and Annexin V^+^/PI^+^ cells were the late apoptotic cells and necrotic cells. The cells were analyzed with a flow cytometer (FACScan; BD Biosciences) equipped with Cell Quest software (BD Biosciences). Cells were sorted into viable cells, early apoptotic cells, and late apoptotic and dead cells. The relative ratio of early apoptotic cells in the experimental groups was compared to that of the control group in each experiment.

### Target prediction

RNAhybrid website (https://bibiserv.cebitec.uni-bielefeld. de/rnahybrid/) was adopted to predict the binding between lncRNA LSINCT5 and miRNA-222. Bioinformatic analysis tool Targetscan (http://www.targetscan.org) can predict the target genes of miRNA-222.

### Luciferase reporter gene assay

The sequence of LSINCT5 (NR_002704.1) RNA was obtained from Pubmed (https://www.ncbi.nlm.nih.gov/pubmed). HEK293T cell line was purchased from American Type Culture Collection (Manassas, VA, USA) and cultured in DMEM media with 10% FBS. Full-length wild-type LSINCT5 and LSINCT5 with the mutated miR-222 binding site were subcloned into pGL3 vector (#E1751, Promega, WI, USA) to construct pLSINCT5-wt and pLSINCT5-mut, respectively. HEK293T cells were co-transfected with pLSINCT5-wt or pLSINCT5-mut and miR-222 mimics or negative control using Lipofectamine 2000 (#11668019, Invitrogen, CA, USA) according to manufacturer’s protocol. Subsequently, cells were transfected with 0.1 μg PRL-TK (TK-driven Renilla luciferase expression vector) as an internal control. Luciferase activities were measured with a dual luciferase reporter assay kit (#E1910, Promega, WI, USA) 48 h after transfection.

### RNA pulldown assay

Biotin-labeled miR-222 (short as miR-222-Bio), biotin-labeled miR-222 (short as miR-222-Mut-Bio) and biotin-labeled negative control (NC) were purchased from Ribobio (Guangzhou, China). Hela cells were purchased from Shanghai Institutes for Biological Sciences, Chinese Academy of Sciences (Shanghai, China) and cultured in DMEM media containing 10% FBS. Hela cells were transfected with miR-222-Bio, miR-222-Mut-Bio or the negative control for 48 h. After that, cells were lysed, and the whole-cell extractions were collected. The lysates were then incubated with Streptavidin Magnetic Beads (#HY-K0208, MCE, NJ, USA) at 4 °C. Two hours later, the biotinylated nucleic acids coated beads were washed to separate the coprecipitated RNA. The samples were then isolated on 10% SDS-PAGE, and target bands were collected for qRT-PCR analysis.

### Western blotting

After transfection and HR treatments, AC16 cells were collected and lysed in 1 × ice-cold RIPA lysis buffer (#P0013B, Beyotime, Shanghai, China). The protein was quantified with the BCA kit (#T9300A, Takara, Dalian, China). A total of 35 μg proteins was separated by electrophoresis on 10% SDS-polyacrylamide gel (PAGE) and transferred onto polyvinylidene difluoride (PVDF) membrane. The membranes were blocked with 1 × Tris-buffered saline with Tween (TBST) containing 5% nonfat milk for 1 h at room temperature and incubated with specific primary antibodies from Abcam (anti-Bcl2, #ab59348, 1:1000; anti-Bax, #ab53154, 1:1000; anti-cleaved caspase 3, #ab2302, 1:1000; anti-PTEN, #ab31392, 1:1000; anti-PI3K, #ab70912, 1:1000; anti-p-PI3K, #ab182651, 1:1000; anti-AKT, #ab8805, 1:1000; anti-p-AKT, #ab8933, 1:1000; anti-β-actin, #ab8227, 1:1000) at 4 ℃ overnight. After wash, the membranes were then incubated with the corresponding secondary antibodies for 2 h at room temperature. Finally, the blot was incubated with ECL plus reagent (#32109, Pierce, Rockford, IL, USA) and visualized using charged-coupled device LAS 4000 (Fujifilm, Valhalla, NY, USA) and photographed. All antibodies were purchased from Abcam (MA, USA) and diluted as suggested by the manufacturer.

### RNA extraction and real-time PCR

Total RNAs were extracted using TRIzol Reagent (#15596026, Invitrogen, Carlsbad, CA, USA) following the manufacturer's protocols after indicated treatment. Then 1 μg total RNAs was reverse transcribed to cDNA using RNA PCR Kit (# RR019A, Takara Biotechnology, Japan) and later used as a PCR template. To detect gene expression, quantitative real-time PCR (qRT-PCR) was performed using an iCycler iQ System with the iQ SYBR Green Super Mix (#170-8882, Bio-Rad, USA) following the manufacturer's protocols. Small endogenous nucleolar U6 snRNA and GAPDH were used as the internal control for normalizing miRNA and mRNAs, respectively. The relative gene expression level wes calculated using the 2^−ΔΔCt^ method. The primers used in this study were β‑actin forward 5′-AAAGACCTGTACGCCAACAC-3′ and reverse 5′-GTCATACTCCTGCTTGCTGAT-3′, U6 forward 5′-CTCGCTTCGGCAG CACA-3′ and reverse 5′-AACGCTTCACGAATTTGCGT-3′, LSINCT5 forward 5′-TCTCCTCC CCTCCAAACACA-3′ and reverse 5′-CTTCCTTGCTTTCATGGGCG-3′, and MiR-222 forward 5′-GCGAGCTACATCTGGCTACT-3′ and reverse 5′-CAGT GCGTGTCGT GGAGT-3′.

### Statistical analysis

All the data are presented as the means ± SD. One-way ANOVA was used to assess the differences between multiple groups, followed by post-hoc multiple comparisons using a Bonferroni post hoc testing. Differences between two groups were analyzed by the Student’s t-test. P < 0.05 was considered statistical significance.

## Results

### LncRNA LSINCT5 expression is significantly upregulated after myocardial ischemia/reperfusion injury

**Fig. 1:**
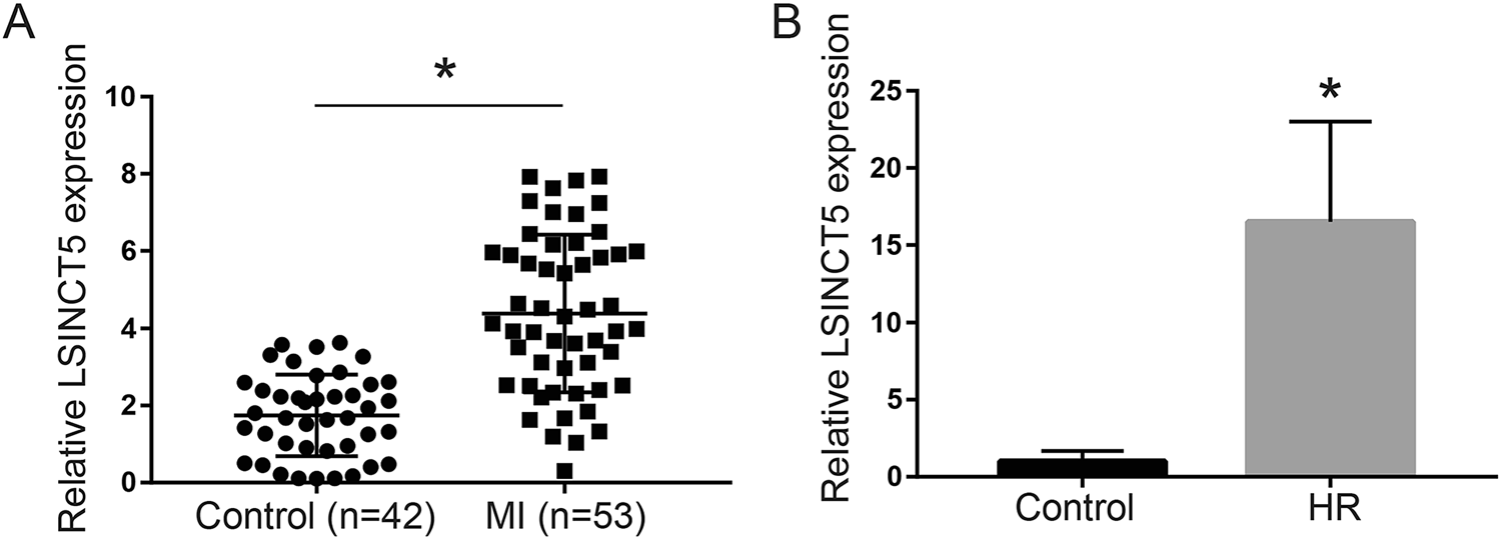
LncRNA LSINCT5 expression is significantly upregulated after myocardial ischemia/reperfusion injury. **A** LSINCT5 levels in the plasma of MI patients (n = 53) and normal controls (n = 42). **B** LSINCT5 levels in HR-induced AC16 cells and control cells were examined by qRT-PCR, with three independent replicates. *P < 0.05 compared with control groups. HR, hypoxia-reoxygenation

To estimate the role of lncRNA LSINCT5 in ischemia‑reperfusion injury, we first evaluated LSINCT5 expression level in MI patients and AC16 cardiomyocyte hypoxia- reoxygenation model using qRT-PCR. As shown in Fig. 1A, LSINCT5 level was significantly higher in MI patients than in the healthy controls (P < 0.05), indicating that LSINCT5 upregulation was likely involved in MI. In addition, LSINCT5 level was upregulated about 16 times following HR treatment in AC16 cells compared with the normal control cells (P < 0.05) (Fig. 1B). These results suggested that LSINCT5 may serve as an important mediator in the process of myocardial ischemia/reperfusion injury.

### LSINCT5 knockdown protects AC16 cells from HR-induced cell death

**Fig. 2:**
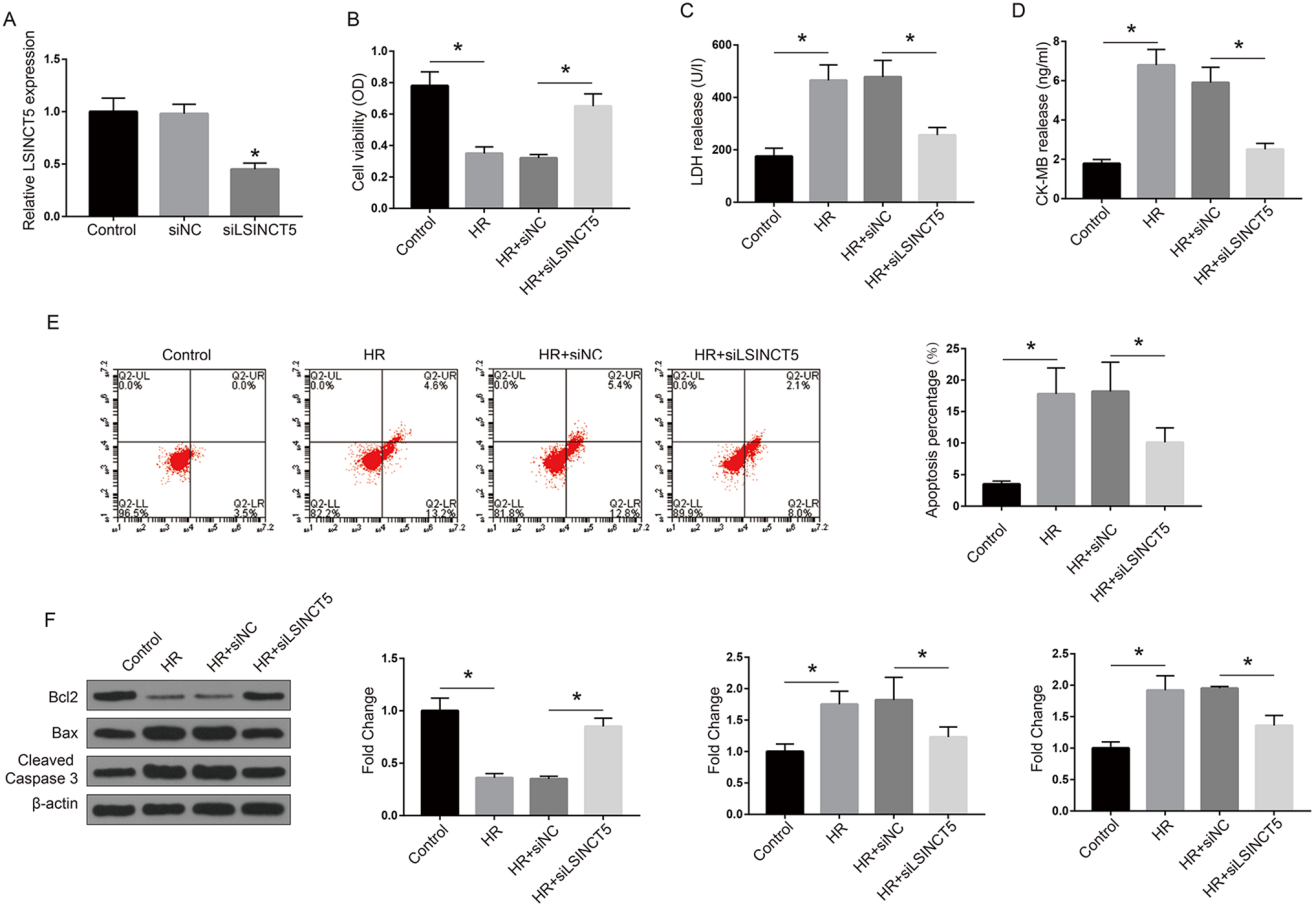
LSINCT5 silencing protects AC16 cells against HR-induced cell death. **A** AC16 cells were transfected with siLSINCT5 and the efficacy was detected by qRT-PCR assay. *P < 0.01, compared with the control. **B** SiLSINCT5 transfection improved cell viability demonstrated by MTT experiment. Levels of myocardial necrosis markers LDH (**C**) and CK-MB (**D**) were evaluated. **E** Apoptosis of AC16 cells under indicated treatment was evaluated by flow cytometry with Annexin V/PI staining. The cells in early and late apoptosis were calculated and shown in histograms. **F** The levels of critical proteins in apoptosis pathways, apoptosis inhibitory protein Bcl-2, pro-apoptotic proteins Bax, and caspase 3, were detected by Western blot. The fold changes of the proteins were quantified and shown in histograms. *P < 0.01. Three independent experiments were performed. *Control* cells under normal culture; *HR* cells with hypoxia/reoxygenation injury treatment; *LDH* lactate dehydrogenase; *CK-MB* creatine kinase isoenzyme

We next performed the knockdown assay to investigate the role of LSINCT5 in HR cell model. The efficacy of siLSINCT5 to knockdown LSINCT5 was confirmed by qRT-PCR. In addition, LSINCT5 level was unchanged in siNC group compared to normal control cells, indicating no damage was caused by transfection agent alone (Fig. 2A). We then examined the viability of AC16 cells after transfection. MTT assay of cell viability demonstrated that LSINCT5 knockdown notably reversed the impaired cell viability caused by HR treatment (P < 0.05; Fig. 2B). We further detected cell death of AC16 by examining the levels of myocardial necrosis markers LDH and CK‑MB in culture medium and cell apoptosis by flow cytometry. The results showed that LSINCT5 silence significantly reduced myocardial enzyme secretion activated by HR (Fig. 2C and D) and significantly decreased the rates of AC16 cell at early and late apoptotic states rates after HR compared with those in siNC transfected cells (P < 0.01; Fig. 2E). Next, we detected the changes of apoptosis-associated proteins, namely the pro-apoptotic proteins Bax and caspase 3 and apoptosis inhibitory protein Bcl-2, by Western blot. The results revealed that Bcl2 was significantly downregulated following HR treatment, while pro-apoptotic proteins Bax and cleaved caspase 3 were upregulated (P < 0.001). Moreover, after LSINCT5 silencing, protein levels of these apoptosis factors exhibited a trend towards recovery (Fig. 2F). These results preliminarily demonstrated that LSINCT5 might participate in the regulation of MIRI-induced cell death.

### LSINCT5 functions as a molecular sponge of miR‑222 in AC16 cells

**Fig. 3:**
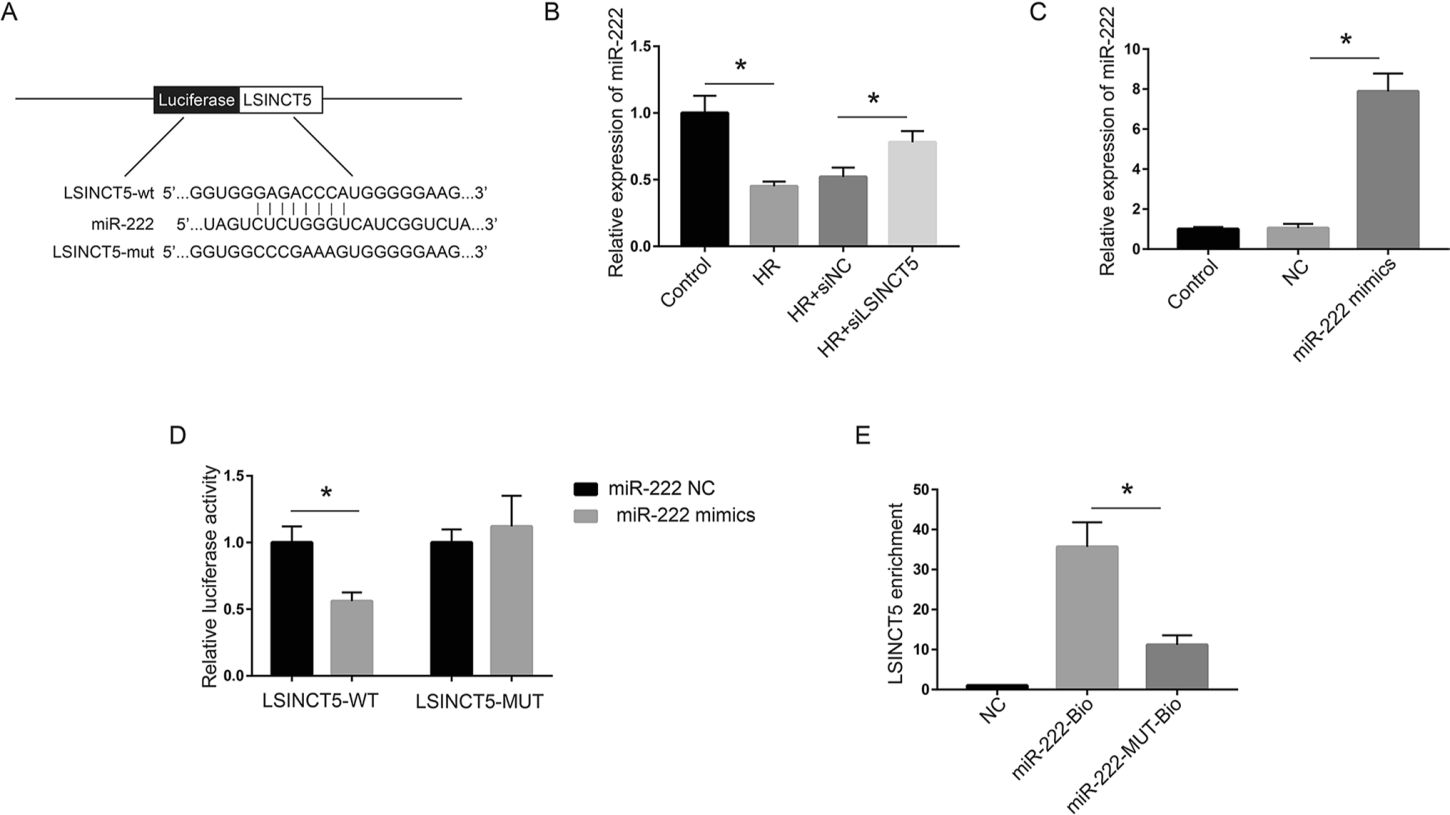
LncRNA LSINCT5 functions as a molecular sponge for miR-222 in AC16 cells. **A** Prediction of the binding region between LSINCT5 and miR-222 by RNAhybrid website. **B** QRT-PCR experiment to evaluate the efficacy of LSINCT siRNA. **C** RT-PCR to evaluate the efficacy of miR-222 mimics and negative control (NC). **D** Luciferase assay was used to test the binding between miR-222 and LSINCT5. HEK293T cells were transfected with LSINCT5-WT or LSINCT5-MUT and miR-222 NC or miR-222 mimics, and the luciferase activity was detected. **E** Pulldown assay was used to evaluate the interaction between LSINCT5 and miR-222-Bio or miR-222-MUT-Bio. *P < 0.05. Three independent experiments were performed. WT, wild type of LSINCT or miR-222; MUT, miR-222 or LSINCT with mutated binding site sequences; Bio, biotin labeled miR-222

Having determined the roles of LSINCT5 in regulating cell viability and apoptosis during MIRI, we next tried to understand the mechanisms involved in these processes. Through screening using bioinformatic tools, we identified micoRNA miR-222 with the highest score and the potential binding sequence (Fig. 3A). Following that, we tested miR‑222 expression in HR cell model after LSINCT5 silencing. qRT-PCR showed a significantly decreased miR-222 level after HR treatment and a notable recovery after LSINCT5 knockdown (Fig. 3B). To further confirm the accuracy of bioinformatic prediction, we investigated the binding ability of miR-222 to 3′UTR fragment of LSINCT5 with a wild-type sequence (LSINCT5-WT) or with a mutated binding region (LSINCT5-MUT) using luciferase reporter gene assay. MiR-222 mimics was used in these experiments, and the efficacy was demonstrated by qRT-PCR (Fig. 3C). The results showed that treatment with miR-222 mimics could significantly decrease the luciferase activity of wild-type LSINCT5, while had no effect on the mutated LSINCT5 reporter plasmid (Fig. 3D), indicating the binding of miR-222 to wild-type LSINCT5 but not to the mutated LSINCT5. We further adopted RNA pulldown assay to directly elucidate the interaction between LSINCT5 and miR-222. As shown in Fig. 3E, transfection of miR-222-Bio but not mutated miR-222 was able to enrich LSINCT5 (Fig. 3E). These results confirmed LSINCT5 as a sponge of miR-222 and demonstrated a possible LSINCT5/miR-222 regulatory axis in HR-induced cell apoptosis.

### LSINCT5 functions through miR-222 to regulate AC16 cell proliferation and apoptosis during HR injury

**Fig. 4:**
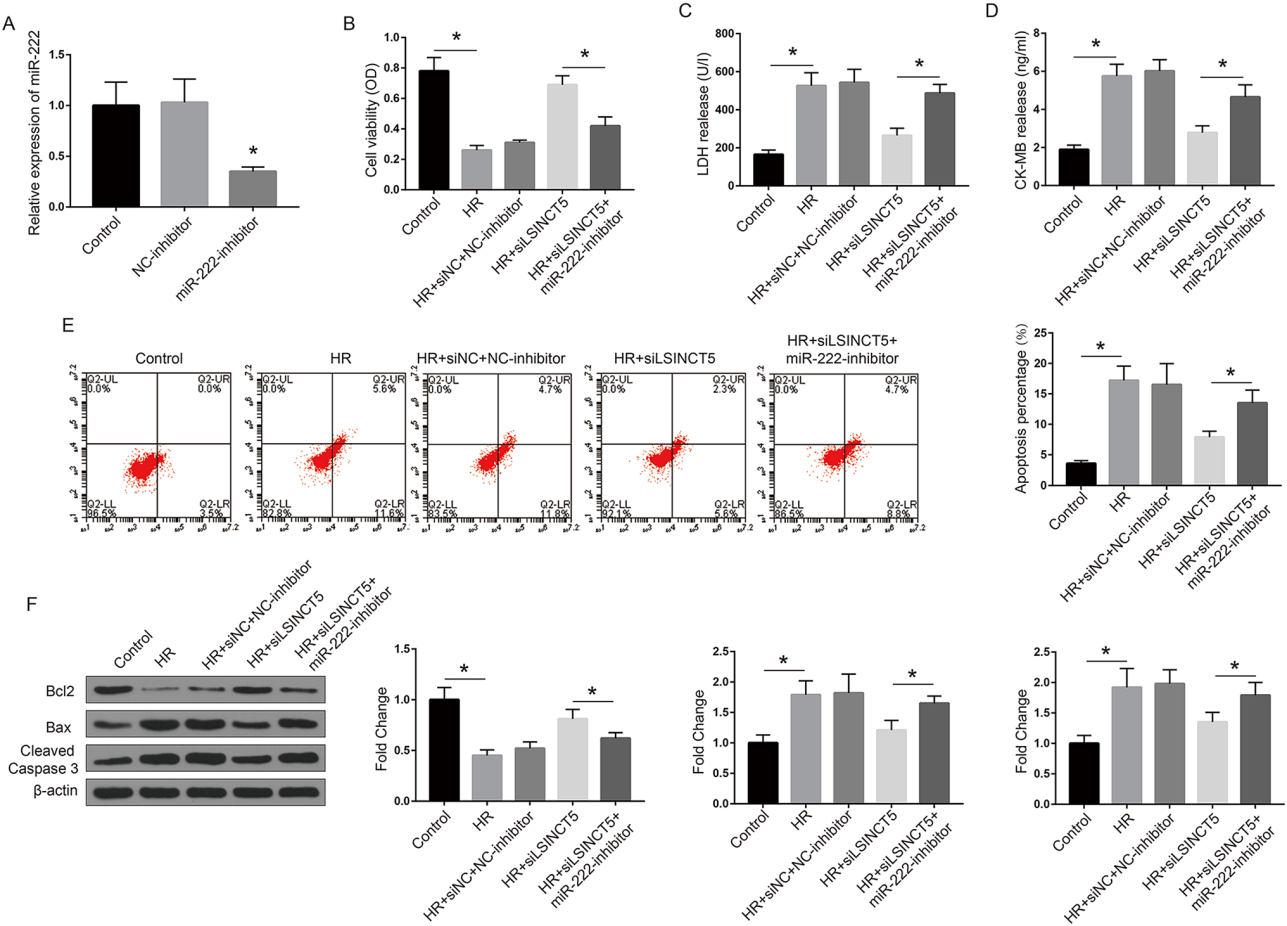
MiR-222 mediates the function of LSINCT5 in HR injury of AC16 cells. **A** AC16 cells were transfected with miR-222 inhibitor. The efficacy was detected by qRT-PCR assay. *P < 0.01, compared with the control. **B**–**F** AC16 cells were with siLSINCT5 alone or together with the miR-222 inhibitor. Cell viability (**B**) was detected by MTT experiment. Levels of myocardial necrosis markers LDH (**C**) and CK-MB (**D**) were detected to evaluate myocardial necrosis. **E** Apoptosis of AC16 cells under indicated treatment was evaluated by flow cytometry with Annexin V/PI staining. The cells in early and late apoptosis were calculated and shown in histograms. **F** The levels of critical proteins in apoptosis pathways, apoptosis inhibitory protein Bcl-2, pro-apoptotic proteins Bax and Caspase 3, were detected by Western blot. The fold changes of the proteins were quantified and shown in histograms. *P < 0.01. Three independent experiments were performed. *Control* cells under normal culture; *HR* cells with hypoxia/reoxygenation injury treatment; *LDH* lactate dehydrogenase; *CK-MB* creatine kinase isoenzyme

We next investigated whether miR-222 mediates the function of LSINCT5 during MIRI. AC16 cells were transfected with siLSINCT5 alone or together with miR-222 inhibitor, followed by HR treatment. The efficacy of miRNA-222 inhibitor was confirmed by qRT-PCR (Fig. 4A). Then we detected the cell viability, myocardial necrosis biomarkers, and cell apoptosis as previously performed. As shown in Fig. 4B, siLSINCT5 transfection significantly upregulated cell viability inhibited after HR, while co-transfection with miR-222 inhibitor reversed the effect of LSINCT5 knockdown. Moreover, secretion of myocardial necrosis biomarkers LDH and CK-MB were reduced with siLSINCT5 transfection compared with HR group, while re-increased after administration of miR-222 inhibitor (Fig. 4C–D). Cell apoptosis alteration assessed by flow cytometry (Fig. 4E) and apoptosis-related biomarkers including Bcl2, Bax and Caspase 3 detected by Western blot (Fig. 4F) demonstrated similar results. These data together indicated that miR-222 is a mediator of LSINCT5 in regulating HR injury of cardiomyocytes.

### lncRNA LSINCT5 affects the PI3K/AKT signaling pathway in HR-induced injury

**Fig. 5:**
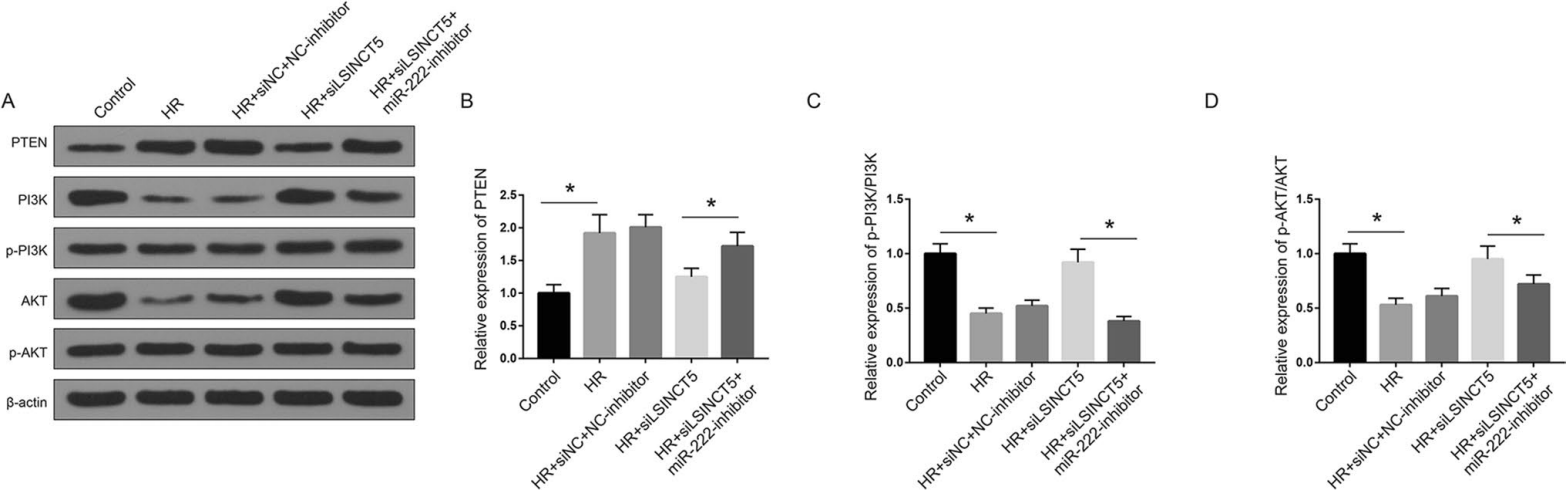
LncRNA LSINCT5 regulates PI3K/AKT signaling through miR-222 in HR injury. **A** Protein expression of PTEN, PI3K, p-PI3K, AKT and p-AKT detected by Western blot. Quantitation of relative protein levels of PTEN (**B**), p-PI3K (**C**) and p-AKT (**D**) were shown in histograms. *P < 0.01. Three independent experiments were performed. *Control* cells under normal culture; *HR* cells with hypoxia/reoxygenation injury treatment; *p-PI3K* phosphorylated PI3K; *p-AKT* phosphorylated AKT

We then elucidated the signaling pathways involved in the LSINCT/miR-222 axis. Considering the significantly altered cell viability after transfection of siLSINCT5 and miR-222 inhibitor, we evaluated the alteration of PI3K/AKT signaling pathway, the critical regulator of cell proliferation. As shown in Fig. 5, expression of PTEN, an inhibitory factor of cell proliferation, was elevated during HR treatment, decreased by silencing LSINCT5, and raised again under co-transfection with miR-222 inhibitor (Fig. 5A and B). Besides, the activation of PI3K and AKT showed an opposite pattern under corresponding treatments. The phosphorylations of PI3K and AKT were obviously downregulated after HR treatment, consistent with impaired cell viability, and recovered after silencing LSINCT5 (Fig. 5C and D). And co-transfection of siLSINCT5 with miR-222 inhibitor led to the significant decline of phosphorylated PI3K and AKT, comparing with siLSINCT5 alone. These results demonstrated that LSINCT5 knockdown activated PI3K/AKT pathway, which could be attenuated by inhibition of miR-222. Hence, we concluded that there might be a LSINCT5/miR-222 axis-regulated PI3K/AKT pathway during myocardial HR injury.

## Discussion

Despite the advancement in therapeutic strategies, cardiovascular diseases, which are usually associated with myocardial ischemia/reperfusion injury after blood reperfusion treatment, remain the leading causes of morbidity and mortality globally [21, 22]. This study indicated lncRNA LSINCT5 plays an important in mediating impaired cell viability and apoptosis by binding to miR-222, and this LSINCT5/miR-222 axis in MIRI may be regulated via PI3K/AKT pathway.

Studies have revealed cell death as a critical factor during multiple pathogeneses of cardiovascular diseases, including MIRI [23]. Moreover, several studies have indicated fewer apoptotic features like DNA laddering in myocardium under ischemia alone while an obvious increment after reperfusion [24, 25]. Our study showed that LSINCT5 level was increased in MIRI patients and the HR-treated AC16 cell model, and LSINCT5 silencing reversed the increased cell death caused by HR treatment, suggesting that increased LSINCT5 level is associated with cell death in MIRI.

Bioinformatics and binding analyses were performed to find microRNAs related to LSINCT5 and confirmed that miR-222 could physically interact with LSINCT5. Further experiments revealed that miR-222 inhibitor reversed the effects of silencing LSINCT5 on cell viability and cell death during MIRI, suggesting that miR-222 may be a mediator of LSINCT5 function.

Recent studies revealed miR-222 being able to regulate hypoxia injury via regulating PI3K/AKT pathway and PTEN expression in viral myocarditis [26, 27]. We further investigated whether PI3K/AKT pathway was disturbed in HR cell model. We showed that PI3K activation was suppressed after HR treatment, which could lead to suppressed cell viability. Moreover, pretreatment with siLSINCT5 and miRNA-222 inhibitor altered the PI3K pathway under HR injury. The PI3K/AKT pathway regulation function of miR-222 might set it from other microRNAs participating in MIRI.

In summary, we studied the function of LSINCT5 in MIRI using MIRI patient and HR-treated cell samples. We found that LSINCT5 was significantly increased in MIRI patients and the HR cell model and increased LSINCT5 level was closely related to the impaired cell viability and apoptosis. We further demonstrated that the function of LSINCT5 was mediated by miR-222 via PI3K/AKT pathway, at least partially. However, whether LSINCT5 knockdown could be a potential therapeutic strategy to treat MI patients needed to be further explored.

## Conclusion

In conclusion, this study demonstrated that lncRNA LSINCT5 is upregulated during myocardial ischemia/reperfusion injury. The possible mechanisms involve a LSINCT5/miR-222 axis and a possible downstream PI3K/AKT pathway. Our study provides supportive evidence for the development of therapeutic strategies for MIRI.

## Supplementary Information

Below is the link to the electronic supplementary material.

Supplementary file1 (DOCX 15 kb)

## Data Availability

The data that support the findings of this study are available on request from the corresponding author Wei Liu (WeiLiupeople@163.com) and Hui Liang (ra1052@163.com) at the Department of Geriatrics, Taihe Hospital, Hubei University of Chinese Medicine, No. 2 Renminnan Road, Shiyan City, Hubei Province 442000, Hubei, China. The data are not publicly available due to their containing information that could compromise the privacy of research participants.
