# Supplementary material for: LncRNA LSINCT5/miR-222 regulates myocardial ischemia‑reperfusion injury through PI3K/AKT pathway
Source: J Thromb Thrombolysis. 2021 Jun 28;52(3):720–9. doi: 10.1007/s11239-021-02506-3 (PMC8568755; doi:10.1007/s11239-021-02506-3)
Supplement: Supplementary file 1 — Supplementary file1 (DOCX 15 kb) [file 11239_2021_2506_MOESM1_ESM.docx]

**Table 1. Other top 20 binding scores of miRNAs that interact with LSINCT5**

| miRNAs | Tot Score | Tot Energy | Max Score | Max Energy |
| --- | --- | --- | --- | --- |
| hsa-miR-6747-3p | 886.00 | -119.95 | 159.00 | -23.68 |
| hsa-miR-6856-5p | 739.00 | -114.51 | 175.00 | -31.03 |
| hsa-miR-6756-5p | 620.00 | -120.72 | 159.00 | -33.03 |
| hsa-miR-3619-5p | 611.00 | -98.24 | 161.00 | -27.13 |
| hsa-miR-4763-3p | 605.00 | -118.49 | 158.00 | -30.98 |
| hsa-miR-1207-5p | 598.00 | -111.02 | 154.00 | -32.89 |
| hsa-miR-4434 | 593.00 | -66.89 | 159.00 | -19.50 |
| hsa-miR-3153 | 593.00 | -86.98 | 153.00 | -29.56 |
| hsa-miR-6736-3p | 592.00 | -91.08 | 157.00 | -29.12 |
| hsa-miR-6510-5p | 588.00 | -97.79 | 158.00 | -32.58 |
| hsa-miR-8080 | 584.00 | -70.57 | 153.00 | -23.64 |
| hsa-miR-3132 | 582.00 | -108.10 | 150.00 | -31.78 |
| hsa-miR-6760-5p | 576.00 | -105.60 | 149.00 | -26.94 |
| hsa-miR-1915-5p | 576.00 | -96.94 | 149.00 | -33.10 |
| hsa-miR-6802-3p | 573.00 | -76.37 | 147.00 | -26.11 |
| hsa-miR-3175 | 569.00 | -81.42 | 146.00 | -22.79 |
| hsa-miR-502-5p | 471.00 | -63.33 | 169.00 | -25.84 |
| hsa-miR-20a-5p | 468.00 | -47.22 | 167.00 | -18.74 |
| hsa-miR-3173-5p | 467.00 | -69.45 | 162.00 | -24.82 |
| hsa-miR-214-3p | 465.00 | -59.16 | 169.00 | -22.71 |
